# The Path to Digestion Is Paved with Repair

**DOI:** 10.1371/journal.pbio.0040307

**Published:** 2006-08-22

**Authors:** Jason Underwood

During the normal course of digesting a human meal, the stomach and subsequent meters of intestinal lining can sustain scratches and physical stresses as food winds through the coiled path. Abrasions are kept to a minimum through the activity of specialized cells that secrete mucus to lubricate the lining.

Now, a study by Katsuya Miyake, Toru Tanaka, and Paul McNeil suggests that the digestive track responds to stresses with a local lubrication response. They used a variety of mucus-producing rodent cells and tissues in combination with several damaging treatment methods to demonstrate that mucus is secreted at the site of injury. At the same time, cells repair their own damaged outer membrane by depositing a “patch” on the injury.

The authors used a simple, yet powerful approach to visualize mucus secretion. Mucus contains glycoproteins, which are modified protein–carbohydrate complexes. Glycoproteins can be monitored using fluorescent versions of proteins called lectins. Since these proteins bind tightly to carbohydrates, the location and intensity of the mucus can be inferred by monitoring the fluorescent glow under a microscope. They also developed an assay to carefully quantify how much mucus was secreted.

Miyake et al. grew gastric surface cells from a rat in culture and subjected them to a variety of stresses. As a general stress, they pushed the cells through a thin syringe needle multiple times, creating perforations in the plasma membrane. The assay revealed that the amount of mucus in the extracellular space increased in a remarkably linear fashion with the number of syringe strokes. Interestingly, without extracellular calcium, mucus secretion was absent. This hinted that the mucus response requires some form of calcium signaling.

These observations led to an intriguing question: do cells respond to injury by switching on generalized secretion and repair or instead have a more specialized mechanism for localizing the mucus response and repairing the wound? To address this question, the researchers used a laser to cause targeted injuries to cells. The response was then visualized with a fluorescent lectin to monitor mucus levels while a special dye in the media monitored the repair response. Without a hole in the cell, the dye is found only on the outside of the cell. If a hole is formed by the laser and is not resealed, the dye can leak through the wound, resulting in a bright intracellular glow.

When the experiment was performed with calcium present, the laser insult resulted in a fast, potent response to the injury site. Mucus is preferentially secreted on the side of the cell where the injury occurred. Also, with calcium present, very little dye accumulates inside the cell during the experimental time course, indicating that the hole is quickly patched. Without extracellular calcium, mucus secretion is absent and the inside of the cell quickly fills with the dye. The researchers' time-lapse movies of these events (see DOI:  10.1371/journal.pbio.0040276.sv001 and DOI: 10.1371/journal.pbio.0040276.sv002) dramatically illustrate this point.

**Figure:**
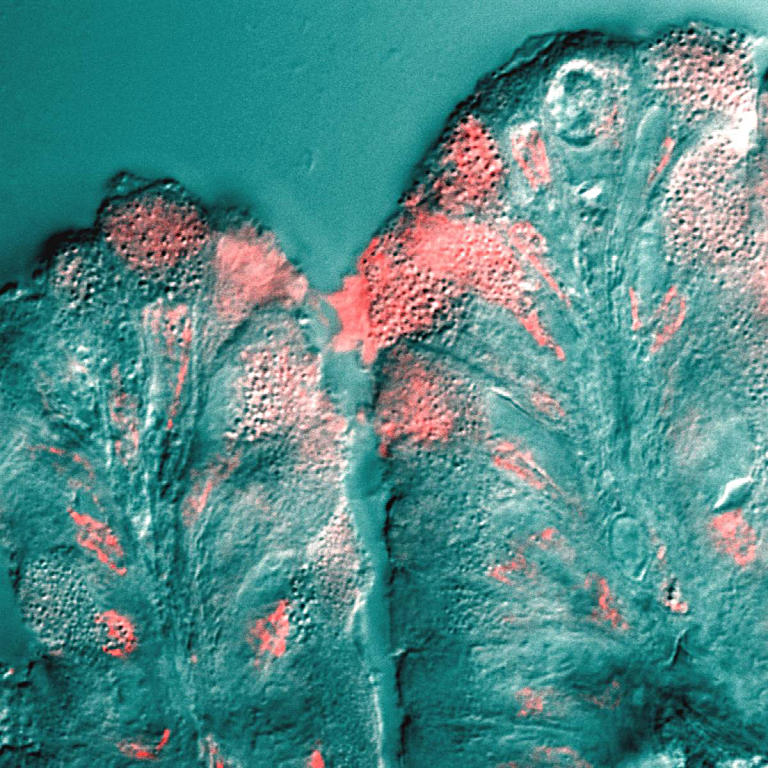
A surface mucous cell bordering on the stomach lumen secretes mucus (pink stain).

Of course, injuries to the mammalian intestinal lining do not result from shear force or lasers, so Miyake et al. approximated the real situation with a different method: using a needle to scratch monolayer-cultured cells or segments of rodent colon. The same localized response was observed; mucus was secreted at the site of injury and resealing occurred. Again, calcium played a key role in both processes. When the authors examined the injury with electron microscopy, they could see that the extracellular membrane architecture changes drastically near the damaged site. Cells form new projections, as if a rough scab is laid down after injury. Again, they uncovered calcium as an essential partner to the repair machinery.

These results show that the cells of the stomach and intestine have an efficient mechanism for repairing ongoing assaults on the digestive tract. The injury itself acts as a signal for both mucus release and an emergency patch response. This adds another checkmark on the growing list of cellular events that function through calcium signaling. With this elegant mechanism in hand, the road ahead is full of important questions: how might one's food intake, genetic disposition or an illness tweak the repair process? And what cellular proteins act as gatekeepers for this process? In any case, the normally dark digestive system has seen a new light.

